# Testing the relation between percentage change and baseline value

**DOI:** 10.1038/srep23247

**Published:** 2016-03-16

**Authors:** Yu-Kang Tu

**Affiliations:** 1 Institute of Epidemiology & Preventive Medicine, College of Public Health, National Taiwan University, Taipei, Taiwan

## Abstract

Testing the relation between percentage change and baseline value has been controversial, but it is not clear why this practice may yield spurious results. In this paper, we first explained why the usual testing of the relation between percentage change and baseline value is inappropriate and then demonstrated how the appropriate null hypothesis could be formulated. We also proposed a simple procedure for testing the appropriate null hypothesis based on the assumption that when there is no relation between percentage change and baseline value, the coefficients of variation for repeated measurements of a random variable should remain unchanged. Two examples were used to demonstrate how the usual testing gave rise to misleading results, whilst results from our simple test were in general consistent with those from simulations. We also undertook simulations to investigate the impact of measurement errors on the performance of the proposed test. Results suggested the type-I error rates increased with the magnitude of measurement errors, whilst the statistical power to detect a genuine relation decreased. The usual approach to testing the relation between percentage change and baseline value tended to yield misleading results and should be avoided.

How to test the relation between change and initial value has been a controversial issue in the statistical literature^1^,^2^,^3^,^4^,^5^. Our previous study reviewed the proposed methods to resolve this controversy and clarified a misunderstanding regarding which methods are more appropriate^1^. However, a related problem with regard to how to test the relationship between percentage change and baseline value remains unresolved^6^,^7^,^8^,^9^. For example, one study found the improvement in thiamine deficiency measured as percentage change in erythrocyte transketolase activity coefficient was strongly correlated with its baseline value in patients admitted with malaria^10^. Therefore, these results seemed to suggest that the improvement in thiamine status depends on the degree of thiamine deficiency at baseline. Another example is a study on using the aldosterone-renin ratio for screening of primary Aldosteronism^11^. In the study, a weak positive correlation around 0.22 was found between plasma aldosterone and plasma rennin activity. Nevertheless, a very strong negative correlation about −0.84 was found between the aldosterone-renin ratio and rennin activity. The authors therefore concluded that because the aldosterone-renin ratio was highly associated with the plasma rennin activity, this ratio was not a renin-independent diagnostic test for screening primary aldosteronism.

However, these studies tested the relation between percentage change and baseline value by correlating two mathematically coupled variables, and it has been shown that this practice is questionable^12^. Suppose *x* is the baseline value, *y* the post-treatment value, and percentage change is defined as (*x* *−* *y*)/*x*. Testing the relation between percentage change and initial value using correlation or regression suffers the same criticism as testing the relation between change and baseline due to *mathematical coupling*^12^,^13^. Mathematical coupling occurs when one variable directly or indirectly contains the whole or part of another, and the two variables are then analysed using correlation or regression^12^. As a result, the statistical procedure of testing the null hypothesis – that the coefficient of correlation or the slope of regression is zero – might no longer be appropriate, and the results need to be interpreted cautiously^1^. However, it is not clear how to obtain the appropriate null hypothesis and how to conduct statistical testing against it.

This article is organized as follows: we first showed how to use the formula derived by Karl Pearson to test the appropriate null hypothesis for testing the percentage change and baseline value under certain assumptions^7^. We then used two examples to illustrate the differences in the results between our proposed approach and the usual practice. Finally, we used simulations to investigate the impact of violations of assumptions owing to measurement errors.

## Pearson’s approximate formula

In 1897, Karl Pearson published a seminal article on “a form of spurious correlation which may arise when indices are used in the measurement of organs”^7^. Pearson showed that if *x*, *y* and *z* are three random variables with the same coefficient of variation, the correlation between *x/z* and *y/z* was around 0.5. Therefore, Pearson argued that “In estimating relative correlation by the hitherto usual measurement of indices, it seems to me that a statement of the amount of spurious correlation ought always to be made” (pp.491). Pearson clearly understood that the potential impact of spurious correlation caused by using ratio indices variables could be enormous in many scientific disciplines, such as biology, medicine, and economics and he frequently came back to discuss this problem throughout his lifetime^14^.

### Is correlation between ratio variables spurious?

Although most authors in the literature agree that a correlation between mathematically coupled variables, such as that between *x*/*z* and *y*/*z*, between (*x* *−* *y*)/*x* and *y*, and between *x* *−* *y* and *x*, is spurious or at least problematic, the explanations in the literature for its spuriousness are ambiguous and diverse. The most commonly used argument or “explanation” is the example of three random variables. For instance, *x*, *y* and *z* are three random variables that follow normal distribution, and the three variables have the same means and standard deviations. Suppose that the pairwise correlations between *x*, *y* and *z* are all zero, and it can be shown that the correlation coefficient between *x/z* and *y/z*, *r*_*x/z*,*y/z*_, is expected to be around 0.5^15^. For the correlation between (*x* *−* *y*)/*x* and *x*, or that between *x* *−* *y* and *x*, is expected to be around 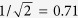. Therefore, the correlations between mathematically coupled variables with shared components cannot be interpreted in the same way as those correlations between variables without shared components. For instance, the strong negative relationship between percentage change in erythrocyte transketolase activity coefficient and baseline activity in patients admitted with malaria may not provide evidence that the improvement in thiamine status depends on the degree of thiamine deficiency at baseline^10^.

In his 1897 paper, Karl Pearson used the binomial expansion to develop a general formula for the approximate correlation between two ratio variables 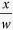 and 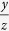 with different numerators and denominators:


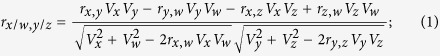


where *V* is the ratio of the standard deviation of a variable over its mean, best known as coefficient of variation, for example, 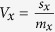 (*s*_*x*_: standard deviation of *x*; *m*_*x*_: mean of *x*). Note that two major conditions need to be satisfied for this formula to be a good approximate of the expected correlation: (1) none of the expected means is zero, and (2) |*V*_*z*_| and |*V*_*w*_| are less than 1 and small^15^. The latter is required for the binomial series to converge.

We can use Pearson’s formula to obtain the expected correlation between percentage change and baseline value, i.e. testing the correlation between *x* and (*x* *−* *y*)/*x*, where *x* and *y* are two measurements of the same variable at baseline and follow-up, respectively. As (*x* *−* *y*)/*x* = 1 *−* *y*/*x*, testing the correlation between *x* and (*x* *−* *y*)/*x* is therefore equivalent to testing the correlation between *x* and *−y*/*x*. The expected correlation between *x* and −*y*/*x* can be shown to be:


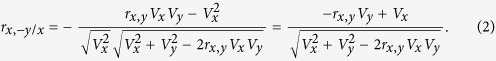


When *x* and *y* also have the same mean and standard deviation and the correlation between *x* and *y*, *r*_*x,y*_ = 0, the expected correlation between *x* and *−y*/*x* is therefore 1/√2 = 0.71. Readers who are familiar with the literature on the relationship between the change and baseline values may find 0.71 familiar, because this is also the expected correlation between *x* *−* *y* and *x* when the correlation between *x* and *y* is zero^1^. This is not surprising: if we take log transformation for these two variables, testing the relationship between *x* and *−y*/*x* becomes testing the relationship between log(*x*) and log(*x*) *−* log(*y*). So the question is why is the correlation 0.71 between *x* and (*x* *−* *y*)/*x* spurious?

### Appropriate null hypothesis

In Karl Pearson’s life time, null hypothesis significance testing or calculation of *p*-values did not exist yet. Nevertheless, in Karl Pearson’s writing, he seemed to suggest that 0.5 is the value that a correlation between two ratio variables such as *x*/*z* and *y*/*z* should be compared to^7^. In the literature, apart from Karl Pearson, Felix Chayes wrote most extensively on the correlations between ratio variables, including a book dedicated to this topic. Most of his works were published in geology and probably due to this reason, are less well known to statisticians^16^. In his book, Chayes gave formula for null correlations between various forms of correlations between ratio variables^17^. For example, for the null correlation between *y*/*x* and *x* (which can still be seen as testing the relationship between percentage changes and baseline values), the equation is given as^17^:


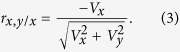


Where *V*_*x*_ and *V*_*y*_ are defined as previously. Apparently, Chayes just like Pearson defined the null correlation by setting the correlations between *x* and *y* in the approximate formula to be zero. Suppose the variables *x* and *y* satisfy the two assumptions for the approximate formula to work properly; nevertheless, whether Eq. 3 can give the appropriate null correlations or not is still debatable. Setting the correlation between *x* and *y* to be zero may not be realistic, because in reality, they are often correlated. For example, the correlation between pre-treatment and post-treatment values is in general positively correlated^18^. Therefore, the equations given by Chayes are unlikely to be suitable for all empirical data.

To obtain the appropriate null correlation (null hypothesis for significance testing), we need to first look at the possible range of correlation between percentage change and baseline value, and clues can be found in the works by Sutherland^19^ and by Bartko and Pettigrew^20^. By starting from the simple scenario regarding the correlation between *y*/*x* and *x*, the expected correlation given by Pearson’s formula is as follows:


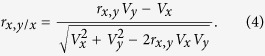


**Figure 1:**
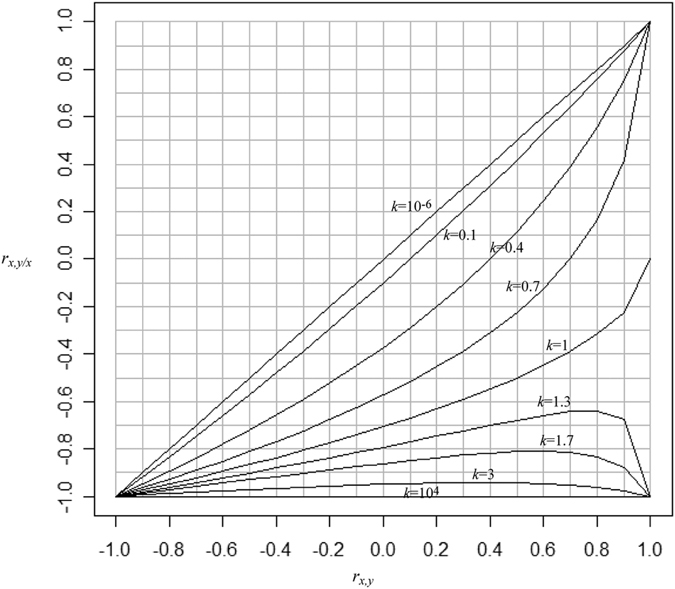
The range of the correlation between *x* and *y*/*x* (*r*_*x,y/x*_) for different values of *k* and *k* is the ratio of the two coefficients of variations for *x* and *y*, conditional on the correlation between *x* and *y* (*r*_*xy*_).

As *r*_*x,y*_ is always less than 1, *r*_*x,y*/*x*_ is more likely to be negative. Suppose *V*_*x*_ = *kV*_*y*_, Eq. 4 can be expressed as:


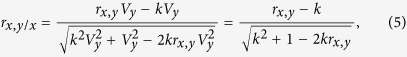


which shows that the values of *r*_*x,y*/*x*_ are affected by *k* and *r*_*x,y*_. When the means of *x* and *y* have the same sign, 0 < *k* < ∞, and as −1 ≤ *r*_*xy*_ ≤ 1, we can plot *r*_*x,y*/*x*_ against *k* and *r*_*x,y*_ (Fig. 1). When *r*_*x,y*_ = −1, *r*_*x,y*/*x*_ will always be −1. When *r*_*x,y*_ increases, the range of *r*_*x,y*/*x*_ becomes greater. When *r*_*x,y*_ = 0, the range of *r*_*x,y*/*x*_ is between 0 (*k* → 0) and −1 (*k* → ∞). When *r*_*x,y*_ is 1, there is a singularity as only three scenarios exist: for *k* = 1, *r*_*x,y*/*x*_ is *undefined*; for any *k* < 1, *r*_*x,y*/*x*_ = 1; and for any *k* > 1, *r*_*x,y*/*x*_ = −1.

From Fig. 1 it is obvious that the plausible range of *r*_*x,y*/*x*_ is not between 1 and −1 as we would expect for a correlation coefficient. Moreover, when *r*_*x,y*_ < 0, *r*_*x,y*/*x*_ will be always smaller than zero. This illustrates that the usual null hypothesis, i.e. that the correlation coefficient is zero, is no longer appropriate due to *r*_*x,y*/*x*_ being constrained by *k* and *r*_*x,y*_; consequently the associated *P*-values are potentially misleading.

The appropriate null hypothesis for the baseline effects on treatment must take into consideration *k* and *r*_*x,y*_. For a given *r*_*x,y*_, the appropriate null hypothesis for the test of *r*_*x,y*/*x*_ is the value of *r*_*x,y*_ when *k* is unity. This may not be obvious at the first sight, so we use a numerical example to explain the rationale. Suppose *x* = (130, 135, 140, 145, 150, 155, 160, 165, 170, 175) and *y* = (104, 108, 112, 116, 120, 124, 128, 132, 136, 140) are two repeated measurements of systolic blood pressure on 10 patients. As *y* = 0.8*x* and *y*/*x* is a vector of 0.8 (i.e. (*x* *−* *y*)/*x* is a vector of 0.2), there is no correlation between *x* and *y*/*x* or between *x* and (*x* *−* *y*)/*x*. Therefore, there is no relationship between percentage change in blood pressure and baseline blood pressure, i.e. the intervention causes the same percentage change in blood pressure across all levels of baseline blood pressure. Note that as *y* = 0.8*x*, *m*_*y*_ = 0.8*m*_*x*_, where *m*_*y*_ and *m*_*x*_ are the means of *y* and *x*, respectively, and also *s*_*y*_ = 0.8*s*_*x*_, where *s*_*y*_ and *s*_*x*_ are the standard deviation of *y* and *x*, respectively. Therefore, 
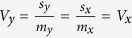
, i.e. 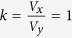. Therefore, if there is no relation between percentage change and baseline value, the coefficients of variation, *V*, would be expected to remain unchanged, and a simple way to evaluate the association between percentage change and baseline value is to evaluate the ratio of the two coefficients of variation: 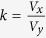. When *k* is close to 1, this suggests no association; when *k* > 1 or *k* < 1, there may be a positive or inverse relation between percentage change and baseline value, respectively.

In this hypothetical example, the correlation between *x* and *y* is unity, but in reality due to biological fluctuations in blood pressure or heterogeneous responses of patients to the interventions, the correlation between *x* and *y* is less than unity. However, if the change in blood pressure is indeed in proportion to the baseline value, i.e. there is *no* relationship in the percentage change and baseline value, *k* should remain unity. Therefore, the appropriate null hypothesis for testing the correlation between *x* and *y*/*x* can be expressed as:


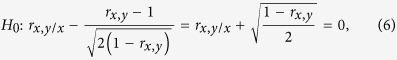


versus


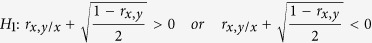


where *r*_*x,y*,*x*_ is the correlation between *x* and *y*/*x*, and *r*_*x,y*_ is the correlation between *x* and *y*. To test *r*_*x,y*/*x*_, we therefore need to know *r*_*x,y*_ and take it into consideration.

Now suppose there is a positive relationship between percentage change and initial blood pressure, i.e. greater percentage reduction in blood pressure can be achieved in patients with greater baseline blood pressure. The hypothetical values of *x* remain unchanged, but the hypothetical values of *y* = (104, 108, 112, 108.75, 112.5, 116.25, 120, 115.5, 119, 122.5), in which the first three values of *y* are equal to 0.8*x*, the middle four equal to 0.75*x* and the last three equal to 0.7*x*. The mean of *x*, *m*_*x*_ = 152.5, and the standard deviation of *x*, *s*_*x*_ = 15.14. The mean of *y*, *m*_*y*_ = 113.85, and the standard deviation of *y*, *s*_*y*_ = 5.87. The ratio of the standard deviation over mean for *x*, *V*_*x*_ = 0.099, and that for *y*, *V*_*y*_ = 0.052. The smaller ratio for *y* is because the change in the standard deviation after treatment in blood pressure is greater than the change in the mean. In other words, when the percentage reduction in baseline values increases with greater baseline values, the decrease in the standard deviation should be greater than the decrease in the mean. On the other hand, when the percentage increment in the baseline values increases with greater baseline values, the increase in the standard deviation should be greater than the increase in the mean.

## Testing the appropriate null hypothesis

As Pearson’s correlation coefficients are not normally distributed, any two correlation coefficients must be transformed before they can be compared. To compare the sample correlation, *r*_*x,y*/*x*_, with the appropriate null value correlation derived, we suggest the use of Fisher’s *z* transformation, according to which 
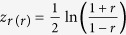
 21. This transformation follows a normal distribution with standard error being 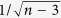, and the following expression for the *z*-test can be used: 
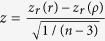
, where *r* is the correlation coefficient of the sample, and *ρ* is the correlation coefficient to be tested against. We test the null hypothesis that *r* *−* *ρ* = 0 by testing 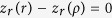. In the next section, we use two examples to illustrate how to undertake the appropriate null hypothesis testing and compare its results to those from computer simulations.

## Example 1: Probing pocket depths in periodontal patients

### Testing the appropriate null hypothesis

A group of 47 periodontal infrabony lesions were treated with a surgical intervention, known as guided tissue regeneration to reduce the periodontal diseases measured by probing pocket depth^22^. The aim of surgery is to reduce the depth of periodontal pocket. The correlation between baseline probing pocket depth (*x*) and the percentage change in pocket depth ((*x* *−* *y*)/*x*, where *y* is the post-treatment pocket depth) was 0.354, suggesting the greater the baseline pocket depth the greater percentage pocket depth reduction was achieved. Conventional significance testing (i.e. where the null hypothesis correlation is assumed to be zero) shows this value to be statistically significant (*P* = 0.015). However, as the correlation between baseline and post-treatment probing pocket depth values, *r*_*x,y*_, was 0.207, the appropriate value for the null hypothesis test, as derived by Eq. 6, is 0.63, not 0. Application of Fisher’s *z* test shows that:


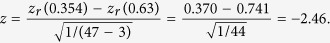


Referring the value *z* = −2.46 to the standard normal distribution yields, for a two-sided test, *P* = 0.014, which is still significant at the 5% level, but however it has an opposite interpretation to the one obtained by the inappropriate hypothesis testing. The inappropriate hypothesis testing seemed to suggest that the greater the baseline pocket depth the greater percentage in pocket depth reduction was achieved. The appropriate hypothesis actually showed the opposite results: the greater the baseline pocket depth the less percentage in pocket depth reduction was achieved. The contradictory results can be explained by the ratios of the standard deviation to the mean for pre-treatment (*x*) and post-treatment (*y*) probing pocket depth: 
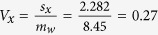
; and 
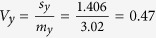
; so 
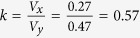
, indicating an inverse relation between percentage change and baseline value. When the treatment yielded the same percentage pocket reduction across all levels of baseline probing pocket depth, *V*_*x*_ should be equal to *V*_*y*_. When the treatment yielded greater percentage pocket reduction in lesions with greater baseline probing pocket depth, *V*_*x*_ should be greater than *V*_*y*_. When the treatment yielded less percentage pocket reduction in lesions with greater baseline probing pocket depth, *V*_*x*_ should be smaller than *V*_*y*_.

### Computer simulations

**Figure 2:**
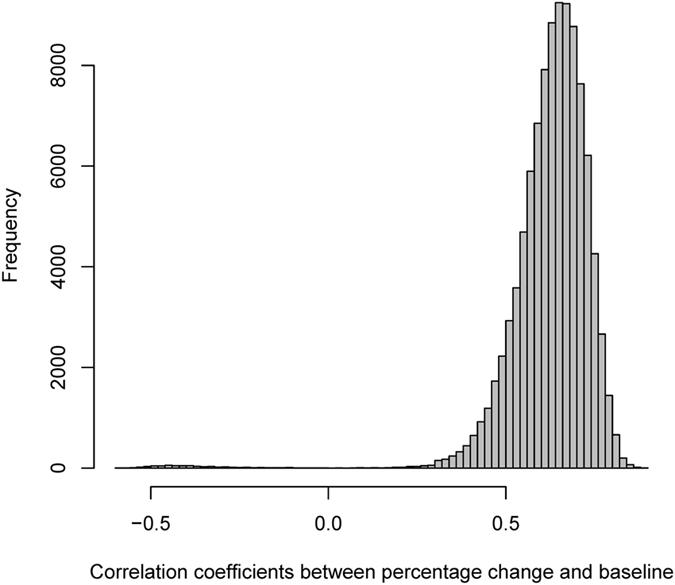
Distribution of correlation coefficient between baseline pocket depth and percentage change in pocket depth based on 100,000 simulations of a group of 47 periodontal infrabony lesions treated with guided tissue regeneration to reduce pocket depth. The median value of the correlation coefficients is 0.64, which is very close to 0.63 derived by Eq. 6.

We now use computer simulations to investigate the robustness of the appropriate null hypothesis approach in this example. Simulations were performed by generating two correlated variables with a sample size of 100,000 each that follow bivariate normal distribution, representing baseline (*x*) and post-treatment values (*y*), using the observed correlation between baseline and post-treatment values from the example data. Alternatively, we could generate 47 simulated pairs of observations and repeat this process 100,000 times. These two approaches would yield the same results. We used the standard deviation of pre-treatment values as the reference to calculate the expected standard deviation of post-treatment values under the null hypothesis that there is no relation between percentage change and pre-treatment values. As the actual sample size *n* is 47, *n* pairs of (*x, y*) were randomly selected from the 100,000 pairs of simulated (*x, y*), and the correlation between *x* and (*x* *−* *y*)/*x* (*r*_*x*,(*x*−*y*)/*x*_) was calculated. This procedure was repeated 100,000 times. All simulations were performed using *R* version 3.2.3. The results from simulations showed that the empirical correlation under the null hypothesis was 0.64 (2.5 and 97.5 centiles: 0.406, 0.778), which is very close to 0.63 derived from Pearson’s formula. Figure 2 is the histogram for the distribution of the 100,000 correlation coefficients. The observed correlation between *x* and (*x* *−* *y*)/*x*, 0.354, is smaller than the 2.5 centile, indicating it is an unusually small correlation, and this confirms the small *p*-value obtained by using the Fisher’s *z*-test. This example showed how conventional null hypothesis testing can be seriously misleading.

## Example 2: CD4 counts in patients on antiretroviral therapy

### Testing the appropriate null hypothesis

In a group of 100 HIV infected subjects on antiretroviral therapy, CD4 counts (cells/mm^3^) were measured at baseline and 4 months later. The aim of antiretroviral therapy was to increase CD4 count. The correlation between baseline CD4 count (*x*) and the percentage change in CD4 count ((*x* *−* *y*)/*x*) was 0.711, suggesting subjects with fewer CD4 positive cells in the blood obtained greater percentage increase in their CD4 count. Conventional significance testing (i.e. where the null hypothesis correlation is assumed to be zero) shows this value to be highly statistically significant (*P* < 0.001). However, as the correlation between baseline and post-treatment CD4 counts, *r*_*x,y*_, was 0.484, the appropriate value for the null hypothesis test, as derived by Eq. 6, is 0.508, not 0. Application of Fisher’s *z* test shows that:


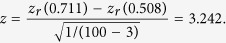


Referring the value *z* = 3.242 to the standard normal distribution yields, for a two-sided test, *P* = 0.001, which is still significant at the 5% level, but is less extreme than the one obtained by the previous conventional hypothesis testing. The results can be explained by the ratios of the standard deviation to the mean for baseline (*x*) and post-treatment (*y*) CD4 count: 
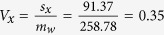
; and 
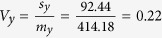
; so 
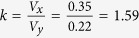
, suggesting a positive relation between percentage change and baseline value. Although the mean CD4 cell count increased after 4 month antiretroviral therapy substantially, the standard deviations showed only very small increase. Therefore, this can be interpreted that the change in CD4 count is almost constant across all level of baseline CD4 count. However, because the increase in CD4 count was almost the same across different levels of baseline CD4 count, this indicated that subjects with smaller CD4 counts actually showed greater percentage increase in CD4 count.

### Computer simulations

**Figure 3:**
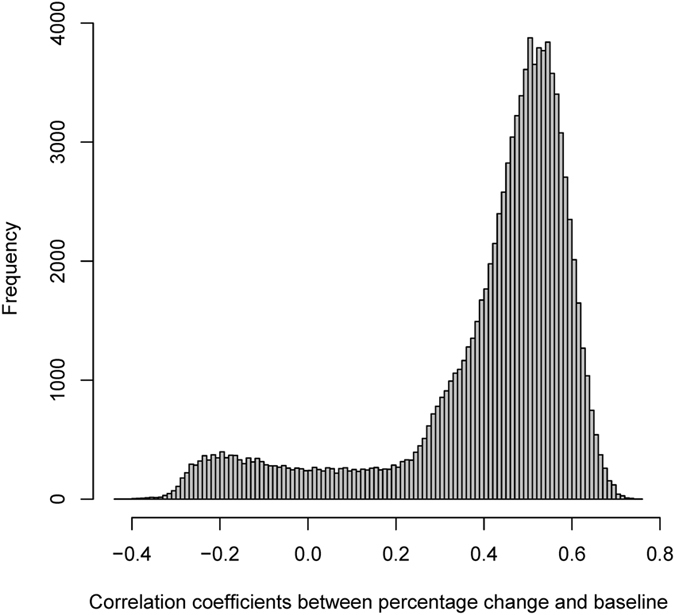
Distribution of correlation coefficient between baseline CD4 count and percentage change in CD4 count based on 100,000 simulations of a group of 100 HIV infected subjects on antiretroviral therapy. The aim of antiretroviral therapy was to increase CD4 count. The median value of the correlation coefficient is 0.479, which is slightly smaller than 0.508 derived by Eq. 6.

The results from simulations showed that the empirical correlation under the null hypothesis was 0.479 (2.5 and 97.5 centiles: −0.222, 0.638), which is slightly smaller than the one derived from Pearson’s formula. Figure 3 is the histogram for the distribution of the 100,000 correlation coefficients. The observed correlation between *x* and (*x* *−* *y*)/*x*, 0.711, is greater than the 2.5 centile, indicating it is an unusually large correlation, and this confirms the small p-value obtained by using the Fisher’s *z*-test.

## Simulations on statistical power of the proposed test and impact of measurement errors

Our proposed test, as expressed in Equation-6, can be viewed as an evaluation of 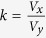; when there is no percentage change, the ratio of a random variable’s standard deviation over its mean, 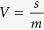, should be constant. This assumes that the random variable can be measured without errors; otherwise even if the true *V* is constant, the observed *V* may change. In reality, variables are often measured with errors, and the magnitude of measurement error may not be proportional to the true value, giving rise to misleading impression that there is a positive or inverse relation between percentage change and baseline value. We therefore undertook simulations by first setting up a random variable *X*, for which the mean is 100 and the standard deviation is 10. The follow-up variable *Y* = 0.8*X*, i.e. the percentage change is 20% and constant across all levels of X, i.e. there is no relation between percentage change 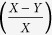 and *X*. We then added measurement error, *e*, for which the mean is fixed at zero but the standard deviation changed from 1 to 5, to both *X* and *Y*, i.e. *x* = *X* + *e*_*X*_ and *y* = *Y* + e_*Y*_, where the standard deviations of *e*_*X*_ and *e*_*Y*_ are equal. Five different sample sizes, 20, 50, 100, 200, and 500, were specified in the simulations. The aims of these simulations are to evaluate the impact of *e* on the type-I error rates of the proposed test, when the true *k* = 1. As the magnitude of *e* is assumed to be independent of both *X* and *Y*, this would likely increase the probability to detect a spurious relation between percentage change and baseline value. We also investigated the type-I error rates of the usual approach by correlating percentage change with the baseline value. We then undertook further simulations by assuming a genuine relation between percentage change in *X* and *X* by specifying (1) *Y* = 0.8*X* *−* 0.001*X*^2^; (2) *Y* = 0.8*X* *−* 0.002*X*^2^; and (3) *Y* = 0.8*X* *−* 0.003*X*^2^ and added *e* to both *X* and *Y*, i.e. *x* = *X* + *e*_*X*_ and *y* = *Y* + *e*_*Y*_, where the standard deviation of *e*_*X*_ and *e*_*Y*_ are equal.

**Figure 4:**
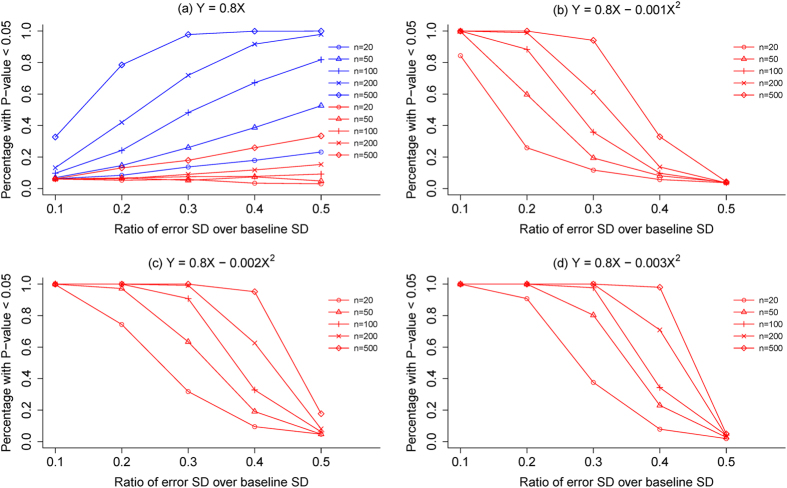
Results of simulations for type-I error rates and statistical power of the proposed test under different assumptions for the ratio of standard deviation of measurement error to the standard deviation of true baseline value for samples of varying sizes: (**a**) the type-I errors of the proposed test (in red lines) and the usual approach to testing the relation between percentage change and baseline value, when the follow-up value Y and baseline value X is Y = 0.8X; (**b**) the statistical power for the proposed test when Y = 0.8X *−* 0.001X2; (**b**) the statistical power for the proposed test when Y = 0.8X *−* 0.002X2; (**b**) the statistical power for the proposed test when Y = 0.8X *−* 0.003X2.

Results of our simulations showed that when there is no relation between percentage change and baseline value, the type-I error rates increased with the standard deviation of *e* and also increased with the sample sizes (Fig. 4a). The type-I error rates of our proposed test increased from 5% to around 30% (red lines in Fig. 4a), but the type-I error rates of the usual approach increased nearly to 100% (blue lines in Fig. 4a). This indicated that the usual approach would very often show a spurious relation between percentage change and baseline value, when there is none. When there is a genuine relation between percentage change and baseline value, (Fig. 4b,c,d), measurement error reduced the statistical power of our proposed test as expected; the greater the measurement error the less likely for the proposed test to correctly detect such a relation. These simulations suggest that adjustment of measurement error should be considered before our proposed test being applied, especially when the magnitude of the error is relatively substantial.

## Discussion

In this article, we demonstrate how to derive the appropriate null hypothesis for the correlation between percentage change and baseline value using Pearson’s approximate formula, and how to test the observed correlation against the appropriate null hypothesis. Results from simulations are close to those obtained from our proposed test. Small discrepancy is expected since in simulations, we assumed a bivariate normal distribution between baseline and follow-up values. Occasionally some improbable negative values were generated, and this occurred more often in Example Two than in Example One, yielding greater discrepancy for the former. From a theoretical point of view, if indeed a treatment can cause percentage change, the post-treatment values may not follow a normal distribution: it could be either left or right skewed, if the pre-treatment values follow a normal distribution. Pearson’s formula provides an approximate correlation coefficient for being used as the null hypothesis, and it requires that the ratio of the variable’s standard deviation over its mean is small. For both simulations and Pearson’s formula to provide reasonable results, the means of pre- and post-treatment values should be farther away from zero. Our simulations also showed when the measurement errors are substantial, adjustment of the measurement errors to obtain appropriate estimates of the standard deviations of baseline and follow-up values may be required to reduce the probability of type-I error and increase the statistical power to detect a genuine relation between percentage change and baseline value.

In previous literature, discussions of percentage changes tended to focus on its performance as an effect size for evaluating the differences in changes from baseline between two groups^23^,^24^,^25^. For instance, Vickers undertook simulations to compare differences in statistical power between change scores, percentage change scores and the analysis of covariance (ANCOVA) and found percentage change scores had a relatively poor statistical power^23^. Regressing the follow-up value *y* directly on *x* yields ANCOVA model: 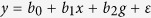, where *b*_0_ is the intercept, *b*_1_ the regression coefficient for *x*, *b*_2_ regression coefficient for the dummy group indicator variable *g*, and the residual error term *ε*; if *b*_1_ is significantly greater or smaller than zero, this seems to indicate that *y* is correlated with *x*. As *y* and *x* are two repeated measurements, it is anticipated that they are more or less correlated. A related model is regressing change score *x* *−* *y* on *x*, such as 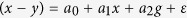. It has been shown that *a*_1_ = 1 − *b*_1_ and *a*_2_ = −*b*_2_, i.e. these two models are just different parameterizations of the same model^26^. A significant regression coefficient *b*_1_ may suggest that the percentage change is constant across all levels of *x*, as how we set up our simulations for Fig. 4a.

In conclusion, the usual approach to testing the relation between percentage change and baseline value tended to yield unacceptable type-I error rates and misleading results; it therefore should be avoided. Our proposed test is simple and easy to implement when certain conditions are met, and the example R code can be found in the online Appendix.

## Additional Information

**How to cite this article**: Tu, Y.-K. Testing the relation between percentage change and baseline value. *Sci. Rep*. **6**, 23247; doi: 10.1038/srep23247 (2016).

## Supplementary Material

Supplementary Information
