# Supplementary material for: Testing the relation between percentage change and baseline value
Source: Sci Rep. 2016 Mar 16;6:23247. doi: 10.1038/srep23247 (PMC4793189; doi:10.1038/srep23247)
Supplement: Supplementary Information [file srep23247-s1.pdf]

Online supplementary materials for "Testing the relation between percentage change and baseline value"

Yu-Kang Tu

Institute of Epidemiology & Preventive Medicine, College of Public Health, National Taiwan University, Taipei, Taiwan

R code for simulation

```
## simulations for Laurell 1994 percentage change and baseline
# x=pre-treatment ppd
# y=post-treatment ppd
library(MASS)
nitn<-100000
nsim<-47
rxy<-0.207
rrx<-0.354
sdx<-2.282
# the observed sd of y is 1.406. Under the null hypothesis
# that there is no relation between percentage change and
# baseline, the expected sd of y is 2.282*(3.02/8.45) = 0.816
sdy<-0.816
mua<-c(8.45,3.02)
sigma<-
matrix(c(sdx*sdx,rxy*sdx*sdy,rxy*sdx*sdy,sdy*sdy),nrow=2)
m<-mvrnorm(100000, mua, sigma)
param1<-c(1:nitn)
param2<-c(1:nitn)
for(i in 1:nitn){
s<-sample(1:nitn,nsim)
smc<-m[s,]
param1[i]<-cor(smc[,2]/smc[,1],smc[,1])
param2[i]<-cor((smc[,1]-smc[,2])/smc[,1],smc[,1])
}
p1<-length(param1[param1>=-0.354])
p1<- 2*p1/nitn
p2<-length(param1[param2<=0.354])
p2<- 2*p1/nitn
hist(param2,col="grey",main="distribution of correlation
coefficients",xlab="Correlation coefficients between
percentage change and baseline",breaks=100)
quantile(param1, c(.025, .5, .975))
quantile(param2, c(.025, .5, .975))


## simulations for CD4 count percentage change and baseline
# x=pre-treatment cd4
# y=post-treatment cd4
library(MASS)
nitn<-100000
nsim<-100
rxy<-0.4835
rrx<-0.7109
sdx<-91.374
# the observed sd of y is 92.44. Under the null hypothesis
# that there is no relation between percentage change and
# baseline, the expected sd of y is
# 91.374*(414.18/258.78) = 146.245
```

```
sdy<-146.245
mua<-c(258.78,414.18)
sigma<-
matrix(c(sdx*sdx,rxy*sdx*sdy,rxy*sdx*sdy,sdy*sdy),nrow=2)
m<-mvrnorm(100000, mua, sigma)
param1<-c(1:nitn)
param2<-c(1:nitn)
for(i in 1:nitn){
s<-sample(1:nitn,nsim)
smc<-m[s,]
param1[i]<-cor(smc[,2]/smc[,1],smc[,1])
param2[i]<-cor((smc[,1]-smc[,2])/smc[,1],smc[,1])
}
p1<-length(param1[param1>=0.7109])
p1<- 2*p1/nitn
p2<-length(param1[param2<=0.7109])
p2<- 2*p1/nitn
hist(param2,col="grey", xlab="Correlation coefficients between
percentage change and baseline",main="distribution of
correlation coefficients",breaks=100)
quantile(param1, c(.025, .5, .975))
quantile(param2, c(.025, .5, .975))
```
